# Clinical variables and magnetic resonance imaging‐based radiomics predict human papillomavirus status of oropharyngeal cancer

**DOI:** 10.1002/hed.26505

**Published:** 2020-10-07

**Authors:** Paula Bos, Michiel W. M. van den Brekel, Zeno A. R. Gouw, Abrahim Al‐Mamgani, Selam Waktola, Hugo J. W. L. Aerts, Regina G. H. Beets‐Tan, Jonas A. Castelijns, Bas Jasperse

**Affiliations:** ^1^ Department of Radiology The Netherlands Cancer Institute Amsterdam The Netherlands; ^2^ Department of Head and Neck Oncology and Surgery The Netherlands Cancer Institute Amsterdam The Netherlands; ^3^ Department of Oral and Maxillofacial Surgery Amsterdam University Medical Center (AUMC) Amsterdam the Netherlands; ^4^ Department of Radiation Oncology The Netherlands Cancer Institute Amsterdam The Netherlands; ^5^ Department of Radiation Oncology Dana‐Farber Cancer Institute, Harvard Medical School Boston Massachusetts USA; ^6^ GROW School for Oncology and Developmental Biology University of Maastricht Maastricht The Netherlands; ^7^ Department of Regional Health Research University of Southern Denmark Denmark; ^8^ Department of Radiology Amsterdam University Medical Center Amsterdam The Netherlands

**Keywords:** head and neck cancer, human papillomavirus, machine learning, radiomics

## Abstract

**Background:**

Human papillomavirus (HPV)‐positive oropharyngeal squamous cell carcinoma (OPSCC) have better prognosis and treatment response compared to HPV‐negative OPSCC. This study aims to noninvasively predict HPV status of OPSCC using clinical and/or radiological variables.

**Methods:**

Seventy‐seven magnetic resonance radiomic features were extracted from T1‐weighted postcontrast images of the primary tumor of 153 patients. Logistic regression models were created to predict HPV status, determined with immunohistochemistry, based on clinical variables, radiomic features, and its combination. Model performance was evaluated using area under the curve (AUC).

**Results:**

Model performance showed AUCs of 0.794, 0.764, and 0.871 for the clinical, radiomic, and combined models, respectively. Smoking, higher T‐classification (T3 and T4), larger, less round, and heterogeneous tumors were associated with HPV‐negative tumors.

**Conclusion:**

Models based on clinical variables and/or radiomic tumor features can predict HPV status in OPSCC patients with good performance and can be considered when HPV testing is not available.

## 1 INTRODUCTION

Human papillomavirus (HPV) infection is an important factor in the development and disease course of oropharyngeal squamous cell carcinoma (OPSCC).^1^, ^2^ HPV‐related OPSCC has a better progression‐free survival and overall survival after (chemo)radiation treatment than HPV‐negative OPSCC.^3^, ^4^, ^5^ Despite these differences in prognosis and treatment response, HPV‐positive and HPV‐negative OPSCC are currently not treated differently. Only recently, it was shown that cetuximab cannot replace cisplatin in HPV‐positive OPSCC.^6^ Ongoing de‐escalation trials will further elucidate whether HPV‐positive tumors can be treated with less aggressive treatment regimens in the future to reduce treatment‐related toxicity (trial number NCT03952585). This is especially relevant as HPV‐positive OPSCC patients tend to be younger with an associated higher life expectancy than HPV‐negative OPSCC patients.^5^, ^7^, ^8^ Adding to the importance of HPV status of OPSCC is the increasing relative incidence of HPV‐positive OPSCC compared to HPV‐negative OPSCC over the past years despite declining overall age adjusted incidence of head and neck cancer in developed countries. These changes are probably due to a decline in alcohol and especially nicotine abuse combined with an increase in sexual promiscuity with a high risk of HPV transmission.^9^ For these reasons, HPV tumor status is increasingly important and has therefore been included in the most recent eighth edition of the TMN classification.^10^

HPV infection is detected using p16/p53 immunohistochemistry and/or HPV DNA polymerase chain reaction (PCR) on biopsy material.^11^, ^12^ Determination of tumor HPV status from just clinical and/or tumor features extracted from imaging would be ideal, and could possibly reduce the need for time consuming and expensive immunochemistry and PCR techniques. Recent literature showed that tumor biology can be assessed noninvasively in other tumor types using advanced imaging analysis or radiomics.^13^, ^14^ The same approach may be used to determine predictive features for the HPV status in OPSCC. Multiple studies reported that the CT‐based radiomic features, such as shape and homogeneity, are associated with HPV positivity in OPSCC tumors.^15^, ^16^, ^17^ To our knowledge, MRI‐based radiomics to predict HPV status has not been performed previously. Clinical variables associated with HPV‐positive tumors are well known and include male gender, younger age, and less exposure to tobacco and alcohol.^9^ These variables have been used to predict HPV status of head and neck cancer, including OPSCC.^18^, ^19^, ^20^, ^21^

This study aims to assess and compare the ability of clinical variables, MR‐based radiomic features, or a combination of these variables to predict HPV status of OPSCC.

## 2 MATERIALS AND METHODS

This study is approved by the local institutional review board (IRBd18047). Due to the retrospective design, informed consent was waived.

### 2.1 Clinical data

A total of 240 consecutive patients with histologically proven primary OPSCC, treated with CRT (70 Gy radiation with three planned cycles cisplatin‐based chemotherapy [100 g/m^2^]) at our Institute between January 2010 and December 2015, were considered for this study. Patients were excluded when pretreatment MRI of the primary tumor was not available (n = 38), image quality was poor (n = 7), tumors were undetectable on MRI (n = 17), a second head and neck tumor was present (n = 1), or when HPV status of the tumor was missing (n = 24). This resulted in a total of 153 patients eligible for this study.

Age, gender, smoking status, tumor subsite, and TNM classification (TNM seventh edition), were collected for each patient. T‐classification and N‐classification was determined in multidisciplinary consensus based on clinical and radiological information, including MRI, ultrasound staging with fine needle aspiration cytology, and, when available, PET images. Smoking status was classified into the categories nonsmoker, current smoker, and former smoker (quit more than 2 years prior to diagnosis) at the initial visit to the outpatient clinic. T‐classification was dichotomized in low (T1 + T2) or high T‐classification (T3 + T4). N‐classification was dichotomized in node‐positive (*N* > 0) or node‐negative disease (*N* = 0). Differences in clinical variables between HPV‐positive and HPV‐negative tumors were assessed by applying the Fisher exact test and independent *t*‐test for age. *P* values of <.05 were considered statistically significant.

### 2.2 Determination of HPV tumor status

A combination of p16 and p53 immunohistochemistry on tumor biopsy material was performed to determine HPV positivity or negativity of the tumor for each patient. p53 positivity was concluded when at least 80% of the tissue sample showed strong nuclear staining or completely negative. No p53 staining of tumor tissue with positive staining of surrounding normal tissue was regarded as tumor mutation for which p53 positivity was concluded. p16 positivity was concluded when at least 70% of tumor tissue stained positive for p16. A known HPV‐positive tonsil sample, surrounding tissue of the tested biopsy sample and appendix, was used as positive internal and external control. HPV positivity was concluded when tumor biopsy material tested positive for p16 and negative for p53 staining. HPV negativity was concluded when tumor biopsy material tested negative for p16, regardless of p53; see Henneman et al^22^ for further details on the HPV testing scheme.

### 2.3 MRI data

All patients underwent an MRI examination of the primary tumor for pretreatment staging purposes as part of the routine clinical workup. Imaging was performed at 1.5 T or 3.0 T (Achieva, Philips Medical System, Best, The Netherlands) using a standard head and neck coil (SENSE‐NV‐16). The imaging protocol included T1‐weighted (T1W), T2‐weighted (T2W), postcontrast 3D T1W, perfusion, and diffusion‐weighted sequences. Imaging details are summarized in Table 1 and Supplementary Table S1.1. The axial slices of 3D T1W high‐resolution isotropic volume excitation (THRIVE) after gadolinium injection (postcontrast 3DT1W) were used to manually delineate primary tumor volumes. One nonexpert observer (PB, 1 year experience in head and neck diagnosis) manually delineated the tumor volumes (ie, nonexpert delineations), which were verified and corrected by an experienced head and neck radiologist (BJ, 7 years of experience in head and neck diagnosis) (ie, expert‐corrected delineations). The observers were allowed to review other available pretreatment MR imaging sequences and available PET scans as reference to improve delineations.

**TABLE 1:** Postcontrast 3DT1W MRI image acquisition parameters stratified by MRI magnet strength, 1.5 T and 3.0 T

MRI field strength	1.5 T, n = 74	3.0 T, n = 79
HPV+	41	35
Slice thickness (mm)	0.8 to 1.0	0.8
Pixel spacing (mm)	0.4 to 1.0	0.2 to 0.8
Repetition time (ms)	9.4 to 10	4.3 to 5.3
Echo time (ms)	4.6	1.7 to 2.4
Echo train length	60	90
Flip angle (°)	10	10

*Note*: HPV, human papillomavirus; 3DT1W, 3D T1‐weighted.

### 2.4 Radiomic feature extraction

Signal intensities for each individual MRI scan were normalized (with zero mean and unit SD) prior to further analysis to reduce intensity variations between MRI scans obtained from different patients. Image resampling to isotropic voxels of 1.0 mm was performed using B‐spline interpolation. Image discretization was applied to allow quantification of texture images in fixed bin width of five. In total, 1184 radiomic features per patient were calculated from the postcontrast 3DT1W MRI within the primary tumor volumes using the open‐source package PyRadiomics 2.2.0,^23^ which were categorized into the five groups: shape, intensity, texture, wavelet transform, and Laplacian of Gaussian filter. Wavelet features were calculated in seven decompositions and texture coarseness is determined by four levels modifying the Gaussian radius parameter from 0.5 to 2.0 mm, in steps of 0.5 mm. Detailed definitions of the radiomic features can be found elsewhere.^28^

After quality control, features with zero variance were excluded. Stable features were selected using the interclass correlation coefficient with regard to the nonexpert and expert‐corrected tumor delineations and the Mann‐Whitney *U* test in features with regard to the different MRI field strengths. Features with an interclass correlation coefficient greater than 0.75 and a significance level equal to or above .05 in the Mann‐Whitney *U* test were considered stable. From the selected stable features, collinear features (Pearson correlation coefficient > 0.9) were removed, where for each pair the feature that has the largest mean absolute correlation is deleted. The remaining 77 features (see consort diagram in Supplementary Figure S1.1) eligible for radiomic analysis were normalized with zero mean and unit variance for analysis.

### 2.5 Machine learning analysis

From the total of 153 patients, 60% (n = 91) were randomly allocated to a training/validation subset and 40% (n = 62) to a test subset, stratifying for HPV status and MRI magnet strength (1.5 or 3.0 T).

Then, separate logistic regression models^24^ were build based on solely clinical variables (ie, age, gender, smoking status, T‐classification, N‐classification, and subsite of cancer) (clinical model), only radiomic features (radiomic model) and a model where both clinical and radiomic features were combined (combined model). As data from other cancer registries may be missing smoking status and/or TN‐classification, we constructed a combined model without smoking status and/or TN‐classification (see Supplementary Material II).

Feature dimensionality is reduced by applying a sequential backward wrapper feature selection approach (recursive feature elimination). This method obtains the optimal feature set for the given classifier (in this case logistic regression) by iteratively removing the weakest feature assessed by its feature importance score. The optimal set of features is used to train the model.^25^, ^26^

In the training phase, Bayesian optimization was used to obtain optimal hyperparameters employing 1000 iterations of 4‐fold cross‐validation on a 75% (n = 68) training and 25% (n = 23) validation set. During this process, the regularization parameter (*λ*, 0.005‐200), a parameter for the complexity of the model, and the number of features (*k*, 1‐77 [radiomic model] or 1‐86 [combined model]) were tuned based on the four training performances obtained during cross‐validation. Area under the curve (AUC) was calculated as measure of model performance, where the loss function is minimized. The loss function was defined as 1 − mean(AUC) + SD(AUC), where mean(AUC) aims to maximize model performance and SD(AUC) aims to minimize model generalization.^27^, ^28^, ^29^

**FIGURE 1:**
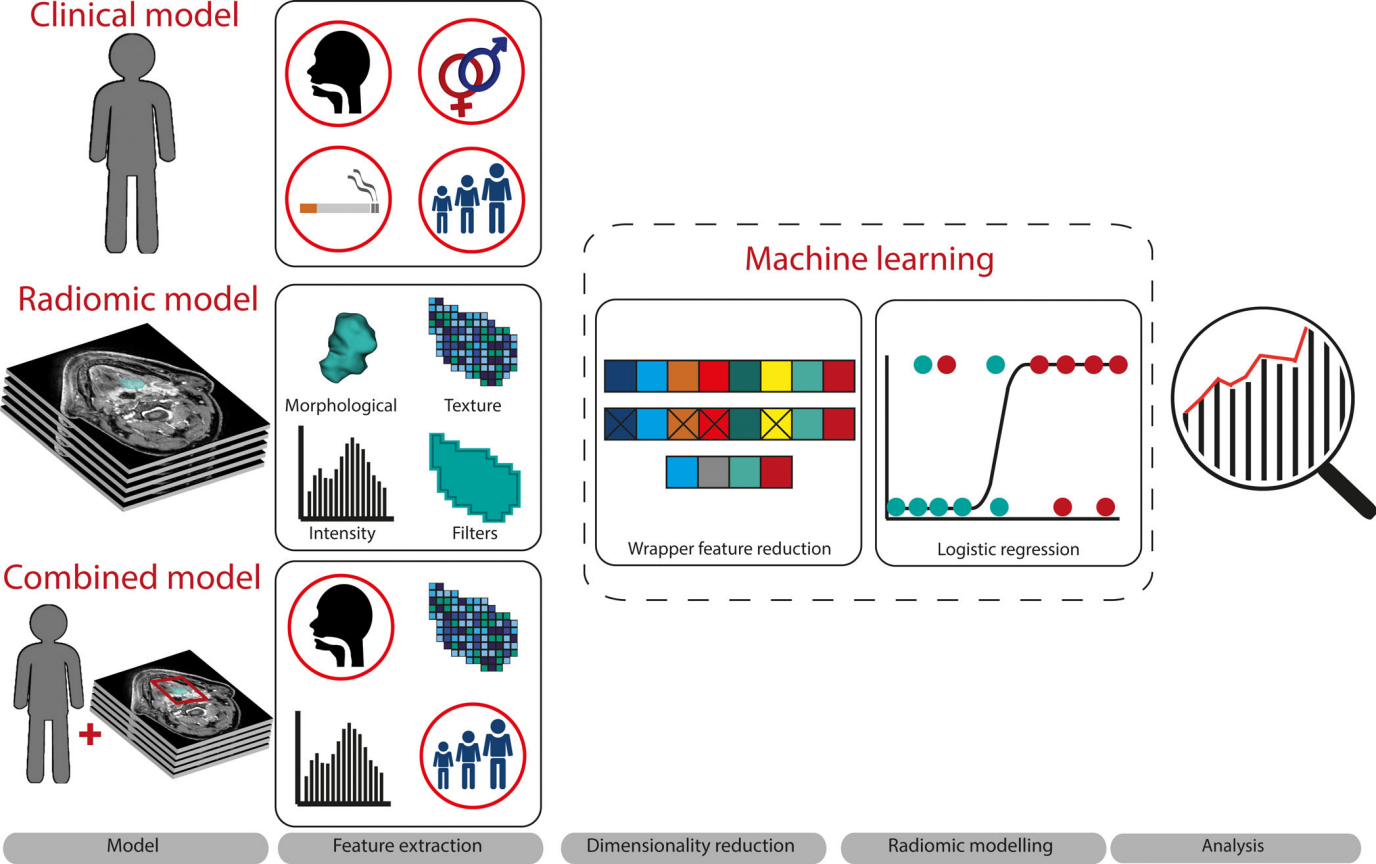
Analysis pipeline. Three models were created to predict human papillomavirus (HPV) status of oropharyngeal squamous cell carcinoma (OPSCC). A clinical model (based on the clinical variables, age, gender, smoking status, T‐classification, N‐classification, and tumor subsite), a radiomic model based on radiomic features, and a combined model based on both clinical variables and radiomic features. Morphological, texture, intensity, and filter‐based radiomic features were computed from within the tumor delineations on the postcontrast 3DT1 MRI images. Feature reduction was performed using the wrapper feature selection approach by recursive feature elimination, resulting in an optimal subset of features as input for the logistic regression models. The three separate models were created using logistic regression analysis on the training subset. Resulting models were tested using bootstrapping with 500 iterations. Model performance on the test set was evaluated using median area under the curve, sensitivity, specificity, accuracy, and its 95% confidence intervals [Color figure can be viewed at wileyonlinelibrary.com]

The optimized hyperparameters obtained in the training phase were then used to verify the predictive model in the test phase, applying bootstrapping on the test subset. Bootstrapping calculated model performance (AUC) of 500 randomly selected samples (with replacement) of the test subset. Median AUC and the 95% confidence interval (95% CI) of these 500 iterations were then calculated to reflect the model performance that can be attained of HPV prediction. All analyses were implemented in python 3.5 and SPSS version 25.0 (SPSS Inc. Chicago). The complete machine learning pipeline is shown in Figure 1.

A clinically applicable nomogram was constructed from the clinical logistic regression model using R software package RMS (version 3.6.3).^30^ Points were assigned to each prognostic variable from the clinical model based on the distribution of the regression coefficients, maximizing sensitivity and specificity for discrimination between HPV‐positive and HPV‐negative tumors. The probability of HPV positivity can be deducted from the sum of these points.

## 3 RESULTS

Table 2 summarizes patient characteristics for the total patient cohort and subgroups stratified by HPV status. The clinical characteristics of the whole patient group have an equal distribution of HPV (n = 77 HPV negative and n = 76 HPV‐positive tumors) and T status (51% patients have T1 + T2 tumors, 49% T3 + T4 tumors). Tumors were mostly located in the tonsils. Patients were categorized as either smoking or nonsmoking, no patients were categorized as former smokers.

**TABLE 2:** Patient characteristics for all patients and subgroups stratified by human papillomavirus (HPV) status of the tumor

Patients	Total n = 153	HPV negative, n = 77	HPV positive, n = 76	*P* value
Age, median y [IQR]	61 [56‐66]	63 [57‐67]	59 [55‐65]	.007^a^*
Male, n (%)	96 (63)	54 (70)	42 (55)	.067^b^
Smoking, n (%)	114 (75)	72 (94)	42 (55)	<.001^b^*
T‐classification, n (%)				<.001^b^*
T1 + T2	78 (51)	25 (32)	53 (70)	
T3 + T4	75 (49)	52 (68)	23 (30)	
N‐classification (N > 0), n (%)	127 (83)	59 (77)	68 (89)	.051^b^
Subsite of cancer, n (%)				
Tonsil	88 (58)	42 (55)	46 (60)	.514^b^
Soft palate	13 (8)	11 (14)	2 (3)	.017^b^*
Base of tongue	48 (31)	20 (26)	28 (37)	.166^b^
Posterior wall	4 (3)	4 (5)	0 (0)	.120^b^

*Note*: The number of patients and its percentage in parentheses is given. Significant values are summarized with an asterisk. Patients were categorized as either smoking or nonsmoking, no patients were categorized as former smokers.

Abbreviation: HPV, human papillomavirus.

a Differences between HPV‐negative and HPV‐positive patient groups calculated with independent *t*‐test.

b Differences between HPV‐negative and HPV‐positive patient groups calculated with Fisher exact test.

OPSCC patients with HPV‐positive tumors were younger (median age: 63 vs 59 year, *P* = .007), less likely to smoke (*P* < .001), and had a lower T‐classification (T1‐T2 vs T3‐T4; *P* < .001) compared to patients with HPV‐negative tumors. For node‐positive disease (*P* = .051) and male gender (*P* = .067), these differences were borderline significant at the 5% level. Tumors of the soft palate (*P* = .017) were significantly more frequent in HPV‐negative tumors.

### 3.1 Performance of logistic regression models

**FIGURE 2:**
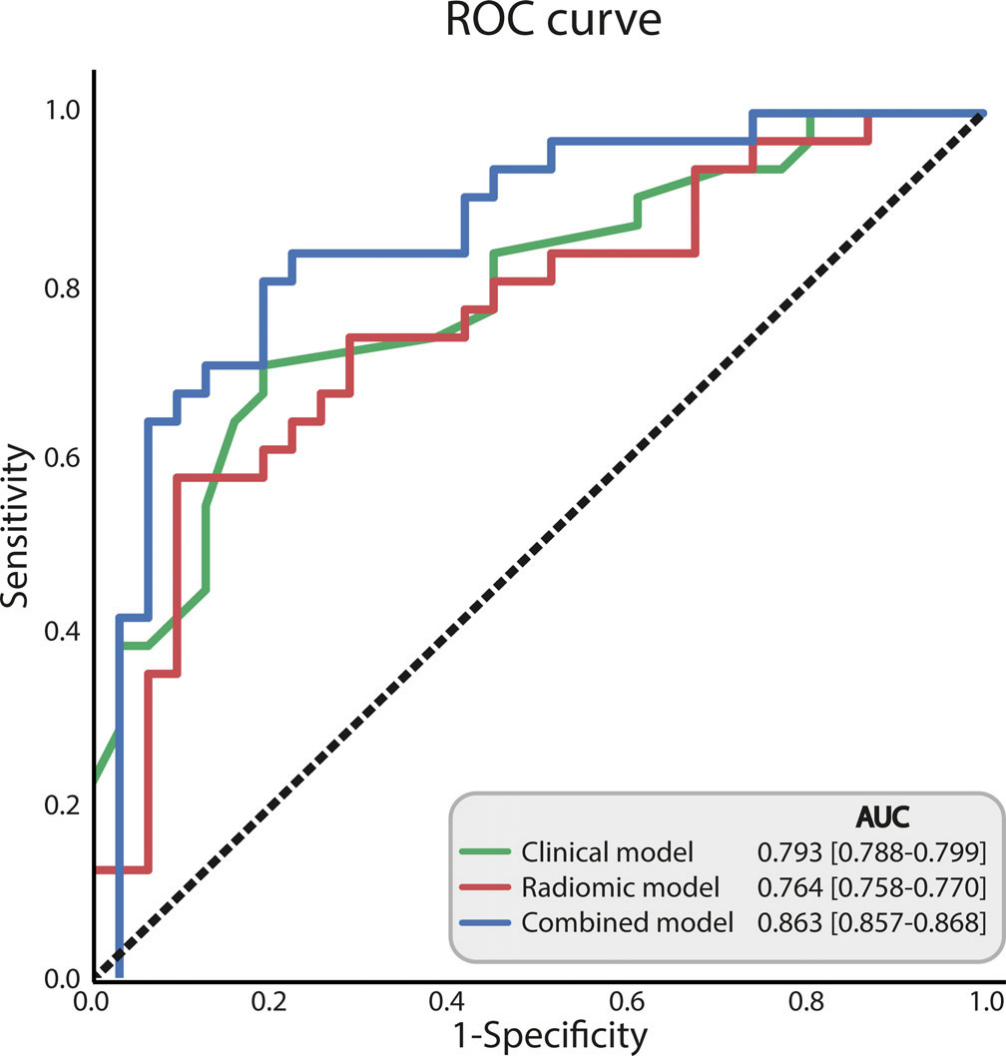
Receiver‐operating characteristic (ROC) curves for prediction of human papillomavirus (HPV) status of the tumor. The combined model had a higher area under the curve (AUC) than the clinical and radiomic model [Color figure can be viewed at wileyonlinelibrary.com]

Performance of the three logistic regression models is summarized in Table 3. All models showed good performance in the prediction of tumor HPV status for the training set (AUC: 0.872‐0.923) and test set (AUC 0.764‐0.871). Figure 2 shows the receiver‐operating characteristic (ROC) curves of the three models. The clinical model (test AUC, 0.794; Sens, 0.71; Spec, 0.81, PPV, 0.79; NPV, 0.74; Acc, 0.76) performed slightly better than the radiomic model (test AUC, 0.764; Sens, 0.76; Spec, 0.71; PPV, 0.72; NPV, 0.75; Acc, 0.73). The combined model had the most favorable performance, outperforming the other models (test AUC, 0.871; Sens, 0.88; Spec, 0.68; PPV, 0.73; NPV, 0.85; Acc, 0.78). Model performance was similar when only smoking status (test AUC, 0.837) or TNM classification (test AUC, 0.873) was omitted from model construction, but drops when both clinical variables were omitted (test AUC, 0.756); see Supplementary Material II for detailed results of the subanalysis.

**TABLE 3:** Model performance of the logistic regression prognostic models for human papillomavirus (HPV) status

Model	Training AUC	Test AUC [CI bootstrap]	Sensitivity [CI bootstrap]	Specificity [CI bootstrap]	PPV [CI bootstrap]	NPV [CI bootstrap]	Accuracy [CI bootstrap]
Clinical	0.872 [0.819‐0.938]	0.794 [0.788‐0.800]	0.71 [0.70‐0.72]	0.81 [0.80‐0.82]	0.79 [0.78‐0.79]	0.74 [0.73‐0.75]	0.76 [0.75‐0.76]
Radiomic	0.885 [0.826‐0.934]	0.764 [0.758‐0.770]	0.76 [0.75‐0.77]	0.71 [0.70‐0.72]	0.72 [0.71‐0.73]	0.75 [0.74‐0.76]	0.73 [0.73‐0.74]
Combined	0.923 [0.868‐0.983]	0.871 [0.866‐0.876]	0.88 [0.87‐0.89]	0.68 [0.67‐0.69]	0.73 [0.72‐0.74]	0.85 [0.84‐0.86]	0.78 [0.77‐0.78]

*Note*: Performance is defined as median AUC with its 95% CI in parenthesis calculated from AUC values of the cross‐validation and bootstrapping for the training and test set, respectively.

Abbreviations: AUC indicates area under the curve; CI, confidence interval; HPV, human papillomavirus; PPV, positive predicted value; NPV, negative predicted value.

### 3.2 Selected features of logistic regression models

**FIGURE 3:**
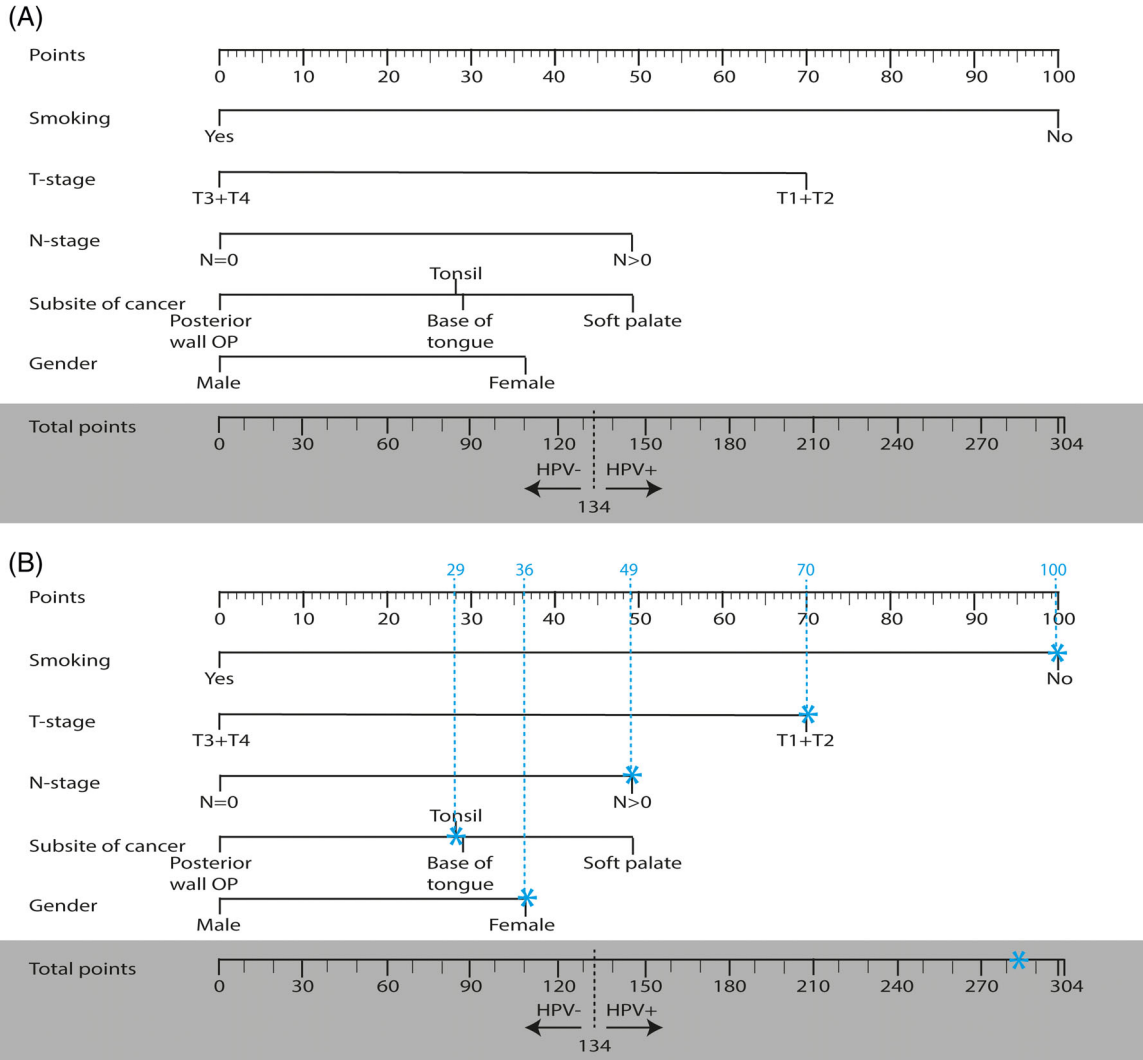
Nomogram for the clinical model to predict human papillomavirus (HPV) positivity. A, Points are given to each clinical by drawing a line between the clinical variable with the “Points” line (top row) ranging from 0 to 100. The sum of all points for the individual clinical variables result in a total score (total points). A total score of ≥134 points is indicative of HPV positivity of the tumor. B, worked example. A nonsmoking female with a T1 tumor of the tonsil region, including node‐positive disease had a total score of 284 points, corresponding to HPV positivity of the tumor [Color figure can be viewed at wileyonlinelibrary.com]

Table 4 summarizes all prognostic variables selected for the three models with their regression coefficients, SE and odds ratios (OR) (95% CI). Selected features were obtained in the training phase, during the last cross‐validation fold, and then used to train the predictive model with the full training dataset. In the clinical model, smoking (OR: 0.47 [0.24‐0.91]), node‐negative disease (OR: 0.69 [0.33‐1.42]), male gender (OR: 0.76 [0.44‐1.34]), tumor located on the soft palate (OR: 0.69 [0.04‐13.15]), and tumor located on the posterior wall of the oropharynx (OR: 0.80 [0.02‐29.97]) were associated with HPV‐negative tumors. A low T‐classification (OR: 1.70 [0.96‐3.03]) and tumor located in the tonsil (OR: 1.24 [0.07‐20.73]) was associated with HPV‐positive tumors. The clinical model is presented in a nomogram in Figure 3, where a cutoff value of 134 points has the maximum sensitivity (76%) and specificity (73%). A sum of points below 134 is indicative of HPV negativity.

**TABLE 4:** Selected features in the radiomic and combined models with regression coefficients ranked from high to low, SEs, and OR (with 95% CI)

Selected feature	Regression coefficient	SE	OR [95% CI]
Clinical model (n = 7)			
Smoking	−0.76	0.17	0.47 [0.24‐0.91]
Low T‐classification	0.53	0.15	1.70 [0.96‐3.03]
Node‐negative disease	−0.38	0.19	0.69 [0.33–1.42]
Subsite of cancer: soft palate	−0.37	0.75	0.69 [0.04–13.15]
Male gender	−0.27	0.14	0.76 [0.44–1.34]
Subsite of cancer: posterior wall of oropharynx	−0.22	0.92	0.80 [0.02–29.97]
Subsite of cancer: tonsil	0.21	0.72	1.24 [0.07–20.73]
Radiomic model (n = 3)			
Shape sphericity	0.16	0.90	1.18 [0.03‐40.59]
Gray‐level co‐occurrence matrix inverse difference moment (Laplacian of Gaussian [2 mm])	0.13	0.11	1.13 [0.73‐1.76]
Kurtosis (wavelet)	0.12	0.22	1.13 [0.48‐2.67]
Combined model (n = 20)			
Smoking^a^	−0.74	0.44	0.44 [0.09‐2.64]
Neighboring gray tone difference matrix busyness (wavelet) (2×)	−0.39	0.88	0.68 [0.02‐21.01]
	−0.21	0.38	0.81 [0.18‐3.61]
Node‐negative disease^a^	−0.33	0.60	0.72 [0.07‐7.53]
Skewness (wavelet)	−0.33	0.32	0.72 [0.21‐2.51]
Shape sphericity^a^	0.33	0.46	1.39 [0.23‐8.35]
Gray‐level co‐occurrence matrix inverse difference moment (Laplacian of Gaussian [2 mm])^a^	0.30	0.12	1.35 [0.86‐2.12]
Subsite of cancer: soft palate^a^	−0.30	0.44	0.74 [0.13‐4.25]
Low T‐classification^a^	0.29	0.55	1.33 [0.15‐11.61]
Kurtosis (wavelet) (3×)^a^	0.29	0.19	1.33 [0.64‐2.77]
	−0.19	0.26	0.83 [0.30‐2.26]
	−0.18	0.38	0.83 [0.19‐3.68]
Neighboring gray tone difference matrix complexity (wavelet)	−0.26	0.00	0.77 [0.77‐0.77]
Maximum (wavelet)	−0.23	0.01	0.79 [0.77‐0.82]
Gray‐level co‐occurrence matrix cluster prominence (wavelet)	−0.23	0.00	0.80 [0.80‐0.80]
Subsite of cancer: tonsil^a^	0.22	0.34	1.25 [0.33‐4.74]
Male gender^a^	−0.22	0.50	0.80 [0.11‐5.71]
Neighboring gray tone difference matrix contrast (2×) (Laplacian of Gaussian [0.5 mm], wavelet)	−0.21	0.10	0.81 [0.55‐1.20]
	−0.18	0.10	0.83 [0.56‐1.24]
Maximum 2D diameter	−0.19	0.04	0.82 [0.71‐0.96]

*Note*: Positive regression coefficients or an OR above 1 indicate a higher likelihood of human papillomavirus (HPV) positive tumor. Negative coefficients indicate a higher likelihood of HPV‐negative tumors.

Abbreviation: CI, confidence interval; OR, odds ratio.

^a^ Features in the combined model that are also included in the clinical or radiomic model.

**FIGURE 4:**
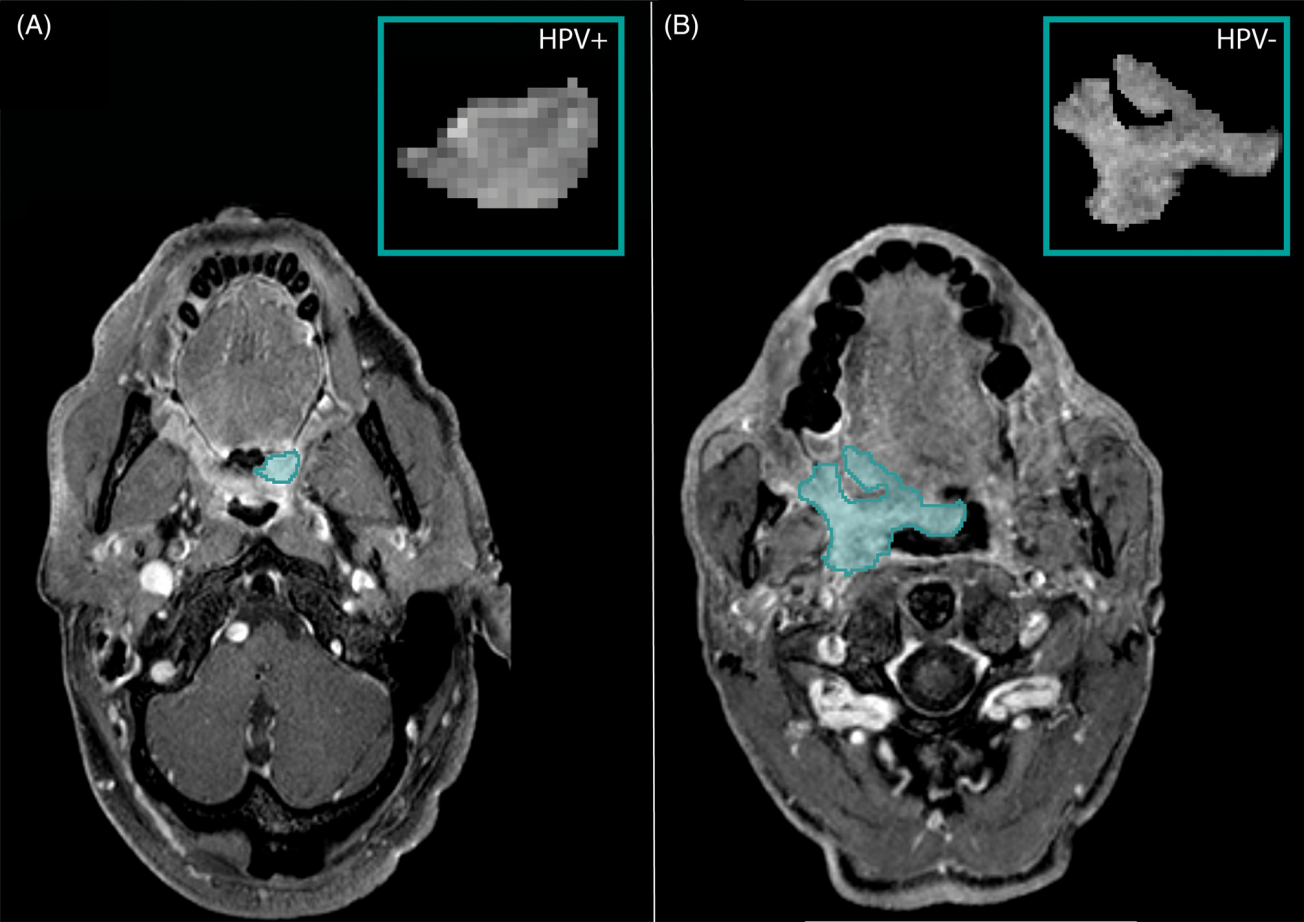
Magnetic resonance image of a patient with human papillomavirus (HPV)‐positive (A) and HPV‐negative (B) tumor status (blue marked area) showing difference in textural appearances. The patient with a HPV‐positive tumor status has a smaller and rounder tumor. Intensity values were less variated and less changes of intensities were visible [Color figure can be viewed at wileyonlinelibrary.com]

Out of the 77 initial radiomic features, three prognostic features were selected in the radiomic model after model construction. Fourteen radiomic features were selected in the combined model, along with six clinical variables that were included in the clinical model. Radiomic features indicated smaller, rounder, more homogeneous, and more regular texture in HPV‐positive tumors. Figure 4 illustrates textural differences between a patient with HPV‐negative and HPV‐positive tumor. The interpretation of all selected radiomic features is summarized in Supplementary Table S1.2.

## 4 DISCUSSION

This retrospective study shows that logistic prediction models based on clinical and/or MR‐based radiomic features are able to predict HPV status in OPSCC with good performance. The model combining radiomic features and clinical variables performed better than separate models based on clinical and radiological features.

The variables included in the clinical model were variables that can be expected to differentiate HPV‐negative and HPV‐positive tumors (ie, smoking status, age, gender, T‐classification, N‐classification, and tumor location). This underscores that the clinical model, besides the good overall performance, is biologically plausible.

The discriminatory MRI features in the radiomic‐based models probably reflect differences in tumor biology between HPV‐positive and HPV‐negative tumors. HPV‐positive tumors are characterized by less‐invasive exophytic growth, nonkeratinizing histopathology, genetic stability, and well‐defined surroundings.^31^ These histopathological differences are likely to be reflected in the selected radiomic features indicating rounder tumors, lower maximum intensity values, and texture homogeneity. Conversely, HPV‐negative tumors are genetically more unstable,^32^ which can lead to focal hypoxia or varying grades of dedifferentiation within a tumor, likely to be reflected in the selected MR features of heterogeneity in the radiomic models.

Although no direct comparison was made, our MR‐based predictive radiomic model suggests similar performance (AUC = 0.76) compared to CT.^16^, ^17^ This suggests that postcontrast 3DT1W MRI and CT reveal, at least partly, similar textural properties relevant for the discrimination of HPV‐positive and HPV‐negative tumors in radiomic analysis. Intuitively, features from MRI and CT should at least be able to characterize tumor size and morphology in a similar way, explaining similar performance. Whether structural MRI or CT is better for determination of HPV status of OPSCC by radiomic analysis is not entirely clear at this point. In our opinion, MRI is preferable over CT for staging and radiomic analysis for OPSCC due to the better soft tissue contrast of MRI in this anatomically challenging area. But in the end, the choice for CT or MRI will largely depend on the preference and experience of the radiologists within the center. The radiomic model presented in this article seems to have better predictive performance compared to fluorodeoxyglucose‐positron emission tomography (FDG‐PET) (AUC: 0.64).^33^ This can be expected as FDG‐PET images are less able to provide textural detail of tumor tissue.

The models in this article are less sensitive (88%) and specific (71%) compared to pathological methods (p16 immunohistochemistry: sensitivity 56‐100%, specificity 79‐93%; DNA PCR: sensitivity: 100% specificity 89% or the combination of latter techniques: Sensitivity and specificity 100%^34^) to determine HPV status of the tumor. However, these pathological methods are expensive and time consuming and are not always available (for instance, in retrospective studies when no biopsy is performed or biopsy/tissue samples are not available), making predictive models based on clinical and/or radiomic features a useful alternative.

This study is, to our knowledge, the largest radiomic study on MRI in head and neck squamous cell carcinomas.^35^ However, our sample size is still quite limited compared to previous studies evaluating CT‐based radiomics.^15^, ^16^, ^17^ Clearly, larger populations, preferably in a multicenter setting, are needed to confirm our findings and create radiomic models that are more generalizable across scanners and populations.

The present study included patients from a single center, without an external cohort to validate our results, which is obviously a recommendation for further work. Another, minor, limitation might be the accuracy of the self‐reporting variables, especially smoking status. This is partly overcome by categorizing smoking status into three robust categories (current‐, former‐, and nonsmoker), where former smokers stopped for at least 2 years prior to diagnosis. Only postcontrast 3DT1W MRIs were used in this study to limit the number of features with our available cases. Other MR sequences might give additional radiomic features for prediction of HPV status and is a topic for further study. In a preliminary study, we included all available MRI sequences, revealing mainly radiomic features from the postcontrast 3DT1W sequence, suggesting that other sequences would not contribute to the eventual predictive models. Finally, time‐consuming manual tumor delineations were used for feature extraction, which introduces interobserver variability. Stable features with regard to delineations were selected to minimize the effect of interobserver variability in the eventual models. Ideally, this interobserver variability should be eliminated. Automated tumor delineation algorithms by, for instance, convolutional neural networks may overcome interobserver delineation variability.^36^ In addition, automated tumor delineation would greatly reduce the workload of manual tumor delineation, making clinical implementation of radiomic analysis more feasible. Another approach would be to use deep‐learning models or other unsupervised machine learning techniques to predict HPV status of head and neck tumors. However, adequate training of these models is challenging due to the relatively small tumors in a large and challenging anatomical area. Radiomic analysis therefore seems to be the most straight forward approach at this point in time.

## 5 CONCLUSION

This study shows that logistic regression models based on clinical variables, MR‐based radiomic features, or a combination of clinical and radiomic features can accurately predict HPV status in OPSCC patients. Although a model based on clinical and radiomic features performs best, the clinical model would be the method of choice due to its ease of implementation. These models have a place in determination of HPV tumor status in settings where tumor biopsy material, tumor samples, immunohistochemistry, and/or DNA polymerase chain reaction techniques are not available. HPV testing is becoming more a routine in hospitals, but not everywhere, especially not in the past when the importance of HPV status of the tumor was not known. Medical images, on the other hand, are widely available due to the advantage of storage capability of medical images for a long time, making it a good alternative to assess HPV tumor status.

## CONFLICT OF INTEREST

H.J.W.L.A. is a stockholder of Sphera and Genospace, outside submitted work. All other authors declare no conflict of interest.

## Supporting information


**Appendix** **S1:** Supporting information
