# Supplementary material for: Clinical variables and magnetic resonance imaging‐based radiomics predict human papillomavirus status of oropharyngeal cancer
Source: Head Neck. 2020 Oct 7;43(2):485–95. doi: 10.1002/hed.26505 (PMC7821378; doi:10.1002/hed.26505)
Supplement: Supplementary file 1 — Appendix S1: Supporting information [file HED-43-485-s001.docx]

**CLINICAL VARIABLES AND MAGNETIC RESONANCE IMAGING-BASED RADIOMICS PREDICTS HUMAN PAPILLOMAVIRUS STATUS OF OROPHARYNGEAL CANCER**

**SUPPLEMENTARY INFORMATION**

**Supplementary material I**

Table S1.1: Imaging details of T1W, T2W and postcontrast 3DT1W MRI.

| **MRI-sequence** | **T1W** | **T2W** | **3DT1W + Contrast** |
| --- | --- | --- | --- |
| **Slice thickness [mm]** | 3-5 | 3-5 | 0.2-1.0 |
| **Pixel spacing [mm]** | 0.4-0.9 | 0.4-0.9 | 0.2-1.0 |
| **Repetition time [ms]** | 180-892 | 1962-6880 | 4.3-10 |
| **Echo time [ms]** | 2.3-10 | 20-90 | 1.7-4.6 |
| **Echo train length** | 1-7 | 10-21 | 60-90 |
| **Flip angle [ᵅ]** | 30-90 | 90 | 10 |

Note: MRI indicates Magnetic Resonance Imaging; T1W, T1-weighted; T2W, T2-weighted; 3DT1W, 3D T1-weighted;

Figure S1.1: Flowchart of included and excluded radiomic features


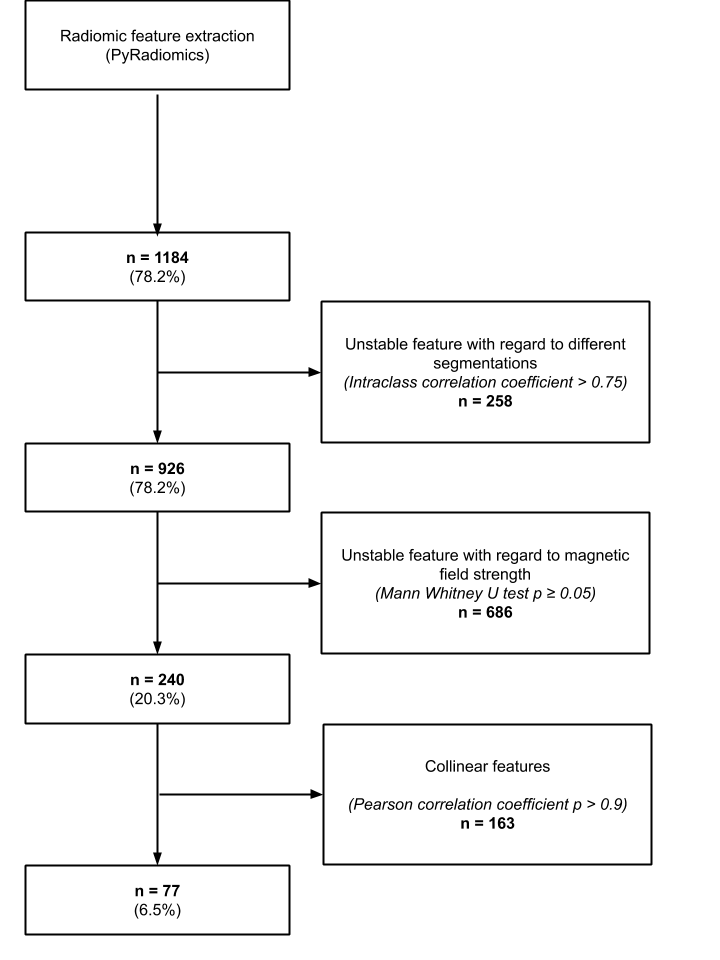


Table S1.2. Interpretation of selected radiomic features. The interpretation is based on HPV positive tumors compared to HPV negative tumors. Features based on specific filters are given in brackets.

| **Category** | **Radiomic feature (filter)** | **Interpretation (HPV positive vs HPV negative tumors)** |
| --- | --- | --- |
| **Shape** | Sphericity | Rounder |
|  | Maximum 2D Diameter | Smaller |
| **Intensity** | Kurtosis (Wavelet) | Less outliers of intensity values |
|  | Skewness (Wavelet) | More symmetrical probability distribution |
|  | Maximum (Wavelet) | Lower maximum intensity value |
|  | Gray Level Co-occurrence Matrix Cluster Prominence (Wavelet) | Less variation of intensity values around the mean |
|  | Gray Level Size Zone Matrix Gray Level Non-Uniformity Normalized (Wavelet) | Greater similarity in intensity values |
| **Texture** | Gray Level Co-occurrence Matrix Inverse Difference Moment (Laplacian of Gaussian (2mm)) | Higher homogeneity |
|  | Neighbouring Gray Tone Difference Matrix Contrast (Laplacian of Gaussian (0.5mm)) | Less local intensity variation |
|  | Neighbouring Gray Tone Difference Matrix Busyness (Wavelet) | Higher homogeneity |
|  | Neighbouring Gray Tone Difference Matrix Complexity (Wavelet) | Less complex, more uniform |

Note: HPV indicates Human Papillomavirus

**Supplementary material II:**

*Sub analysis: HPV prediction in combined models without smoking status and/or TNM-classification.*

Head and neck tumor databases may not include clinical variables like smoking status and TNM-classification. To provide a model that can predict HPV status without smoking and/or TNM-classification, three additional combined models were created using the same methods as described in the main article. One model was created without smoking status, one without tumor classification (T- and N-classification), and, one without both smoking status and tumor classification to predict HPV status of the tumor.

All combined models showed good performance in the prediction of HPV status of the tumor with AUCs ranging from 0.756 to 0.873 in the test set (see table S2.1). The combined models that excluded smoking status (Test AUC: 0.837, accuracy: 0.73) or tumor classification (Test AUC: 0.873, accuracy: 0.80) had a predictive performance that was comparable to a model including those variables (Test AUC: 0.871, accuracy: 0.78). Performance of the combined model lacking both variables decreased (Test AUC: 0.756, accuracy: 0.73) compared to a model were those variables were available.

Table S2.1: Model performance of the logistic regression prognostic models for HPV-status. Performance is defined as median AUC with its 95% CI in parenthesis calculated from AUC values of the cross-validation and bootstrapping for the training and test set respectively.

| **Model** | **Training AUC** | **Test AUC**  **[CI bootstrap]** | **Sensitivity**  **[CI bootstrap]** | **Specificity**  **[CI bootstrap]** | **PPV**  **[CI bootstrap]** | **NPV**  **[CI bootstrap]** | **Accuracy**  **[CI bootstrap]** |
| --- | --- | --- | --- | --- | --- | --- | --- |
| Combined without smoking status | 0.896  [0.834-0.975] | 0.837  [0.831-0.842] | 0.83  [0.82-0.83] | 0.65  [0.64-0.66] | 0.70  [0.70-0.71] | 0.79  [0.78-0.79] | 0.73  [0.73-0.74] |
| Combined without TN-classification | 0.919  [0.882-0.942] | 0.873  [0.868-0.878] | 0.85  [0.84-0.86] | 0.77  [0.77-0.78] | 0.79  [0.78-0.80] | 0.83  [0.83-0.84] | 0.80  [0.79-0.81] |
| Combined without smoking status and TN-classification | 0.885  [0.823-0.934] | 0.756  [0.750-0.763] | 0.76  [0.75-0.77] | 0.71  [0.70-0.72] | 0.72  [0.71-0.73] | 0.75  [0.74-0.76] | 0.73  [0.73-0.74] |

Note: AUC indicates Area under the curve; CI, Confidence Interval; HPV, Human Papillomavirus; PPV, Positive predicted value; NPV, Negative predicted value
